# First record of silk-loving genus *Monteithophila* (Hemiptera, Heteroptera, Plokiophilidae) from Asia, with description of a new species from China

**DOI:** 10.3897/zookeys.1268.180987

**Published:** 2026-02-02

**Authors:** Haoyang Xiong, Zhuo Chen, Hu Li, Wanzhi Cai

**Affiliations:** 1 State Key Laboratory of Agricultural and Forestry Biosecurity, MARA Key Lab of Pest Monitoring and Green Management, College of Plant Protection, China Agricultural University, Beijing 100193, China College of Plant Protection, China Agricultural University Beijing China

**Keywords:** Heissophilinae, Heissophilini, identification key, morphology, Oriental Region, taxonomy

## Abstract

The silk-loving bug *Monteithophila hainanensis***sp. nov**. (Hemiptera, Heteroptera, Plokiophilidae) is described from Hainan, southern China, where it was found on the webs of *Spinathele* sp. (Araneae, Macrothelidae). This new species represents the first record of the genus *Monteithophila* Schuh, Štys & Cassis, 2015 outside Oceania. The new species can be distinguished from its congeners by its smaller body size, head uniformly colored yellowish brown to reddish brown, fore major claws slightly longer than mid ones with no significant difference, and distinct morphology of corial glands on the hemelytra. A key to the species of *Monteithophila* is presented. In addition, the ecological characteristics of the new species and the morphology of the genitalia are briefly discussed.

## Introduction

The family Plokiophilidae is a behaviorally unique and rarely collected group of true bugs, with most of its species being found inhabiting the webs of spiders or embiopterans whereas a few species have also been discovered in soil samples, leaf litter, flight interception traps, or malaise traps (Carayon 1974; Štys 1991; Carpintero and Dellapé 2005; Štys and Baňař 2016; Schuh and Weirauch 2020). To date, a total of nine genera and 23 species have been described (including one Baltic amber fossil genus and one fossil species) (Popov 2008; Xiong et al. 2025). These bugs are tiny (1.2 to 3 mm in size), with many species completing their entire life cycles on webs and records indicating they feed on hosts’ food, eggs, juveniles, weak adults, or other small arthropods thereon (China and Myers 1929; China 1953; Carayon 1974; Luo et al. 2021; Büscher et al. 2023).

The first species of Plokiophilidae, *Arachnophila cubana* China & Myers, 1929, was discovered in Cuba, and although its unique behaviors were recognized as making it difficult to assign to any family within Cimicoidea, it was tentatively placed in Microphysidae out of caution (China and Myers 1929). Due to the homonymy conflict between *Arachnophila* China & Myers, 1929 and *Arachnophila* Salvadori, 1874 (Aves), China (1953) proposed a new name, *Plokiophila* China, 1953, for it, also describing a new genus and species collected from the webs of embiopterans in Trinidad and establishing Plokiophilinae as a subfamily of Microphysidae. Carayon (1961) elevated Plokiophilinae to the family level and proposed the subfamilies Plokiophilinae and Embiophilinae. Štys (1962) supported this taxonomic change and provided a key to Plokiophilidae while describing a new genus and species, *Lipokophila chinai* Štys, 1967, from Brazil (Štys 1967). Carayon (1974) published a monograph of this family, describing a new genus and seven new species from continental Africa and Brazil. Subsequently, several new genera and species of Plokiophilinae have been reported successively, yet the taxonomic system of Plokiophilinae was not revised again until 2015 (Štys 1991; Eberhard et al. 1993; Carpintero and Dellapé 2005; Schuh 2006; Popov 2008). Schuh et al. (2015) published an influential review on Plokiophilinae, in which they downgraded Embiophilinae to a tribe within the subfamily Plokiophilinae based on male and female genitalic structures, proposed the new subfamily Heissophilinae and the new tribe Lipokophilini, established *Monteithophila* Schuh, Štys & Cassis, 2015 and *Paraplokiophiloides* Schuh, Štys & Cassis, 2015, and described three new species. Since then, all taxonomic studies on Plokiophilinae (Štys and Baňař 2016; Henry 2020; Luo et al. 2021; Xiong et al. 2025) have adopted the classification system proposed by Schuh et al. (2015).

The genus *Monteithophila* Schuh, Štys & Cassis, 2015 was established to accommodate two new species from Oceania: *M. fijiensis* Schuh, Štys & Cassis, 2015 from Fiji, and the type species *M. queenslandana* Schuh, Štys & Cassis, 2015 from northeastern Australia (Schuh et al. 2015). The genus was assigned to the subfamily Heissophilinae based on three-segmented tarsi and a short, broad male pygophore that is not tubular.

During our recent field investigations in Hainan, southern China, we collected a number of specimens of a plokiophilid species from the webs of a spider belonging to the genus *Spinathele* Shao, Zhou & Lin, 2025 (Araneae, Macrothelidae). Further examinations revealed that these specimens represent a new species of *Monteithophila*. This new species represents the first record of *Monteithophila* in China and the Oriental Region. An identification key to the species of *Monteithophila* is provided, and the ecological characteristics of the new species, along with the male genitalic structures and distribution of the genus, are briefly discussed.

## Material and methods

Specimens examined in this study were deposited in the Entomological Museum of China Agricultural University, Beijing, China (**CAU**).

External and genital structures were examined using a Nikon SMZ745 stereoscopic microscope. Measurements (in mm) were taken using a Keyence Large depth-of-field microscope (VHX-X1) with Adobe Photoshop 2024. Male genitalia and female abdomen were macerated in 10% potassium hydroxide solution (KOH) at 60 °C for 3 h. Photographs were taken using the Keyence Large depth-of-field microscope (VHX-X1). Ecological photos were taken with a Nikon D5 camera with a LAOWA 100 mm f/2.8 lens. Scanning electron micrographs were prepared using a Regulus 8100 Scanning Electron Microscope at State Key Laboratory for Biology of Plant Diseases and Insect Pests, Institute of Plant Protection, Chinese Academy of Agricultural Sciences, Beijing, China. Figure plates were prepared using Adobe Photoshop 2024. Distribution map was prepared using QGIS Desktop 3.34.11.

The classification system of Plokiophilidae follows Schuh et al. (2015). Morphological terminology mainly follows China and Myers (1929) and Schuh et al. (2015). In terms of measurements, length of body is defined as the straight-line distance from the anterior tip of the head to the posterior end of the abdomen; maximum width of pronotum is defined as the distance between the two posterolateral angles of the pronotum; maximum length of pronotum is defined as the perpendicular distance from the anterior margin of the pronotum to the line connecting the posterolateral angles.

Abbreviations used in the text and figures are as follows:

**a** acus

**ap** articulatory apparatus

**cgs** corial glands

**cp** corial process

**Cu** cubitus

**gps** gland pores

**ic** inner claw

**lp** left paramere

**M** media

**oc** outer claw

**pe** parempodia

**R** radius

**rp** right paramere

**Sc** subcostal

**sv** secondary vein

**ts1** first segment of tarsus

**ts2** second segment of tarsus

**ts3** third segment of tarsus

**1A** first anal vein

## Taxonomy

### Genus Monteithophila

Taxon classificationAnimaliaHemipteraPlokiophilidae

Schuh, Štys & Cassis, 2015

7CF7ED24-E408-5FB7-B3B4-632B967A3315

Monteithophila Schuh, Štys & Cassis, 2015: 4. Type species by original designation: Monteithophila queenslandana Schuh, Štys & Cassis, 2015.

#### Diagnosis.

*Monteithophila* can be distinguished from other genera of Plokiophilidae by the following combination of character states: head lacking elongate neck behind eyes; macrochetae absent on frons, vertex and pronotal collar; tarsi 3-segmented; foreleg with very long major (inner) claw; fossula spongiosa absent on all legs; membrane of hemelytron with three weakly-developed free veins; cuneus absent; pygophore broader at base than apex; parameres symmetric, exposed on dorsal surface, with apices faced anteromedially; all traces of ovipositor lost in females.

#### Diversity and distribution.

The genus previously contained two species from Oceania (Schuh et al. 2015). The new species described herein is distributed in the Oriental Region.

### Monteithophila hainanensis sp. nov.

Taxon classificationAnimaliaHemipteraPlokiophilidae

FD9E9690-0899-5470-B4C5-9D8A47751001

https://zoobank.org/E4327CBE-AE69-4BA5-B6F4-31DC00B53F8A

**Figure 1.**
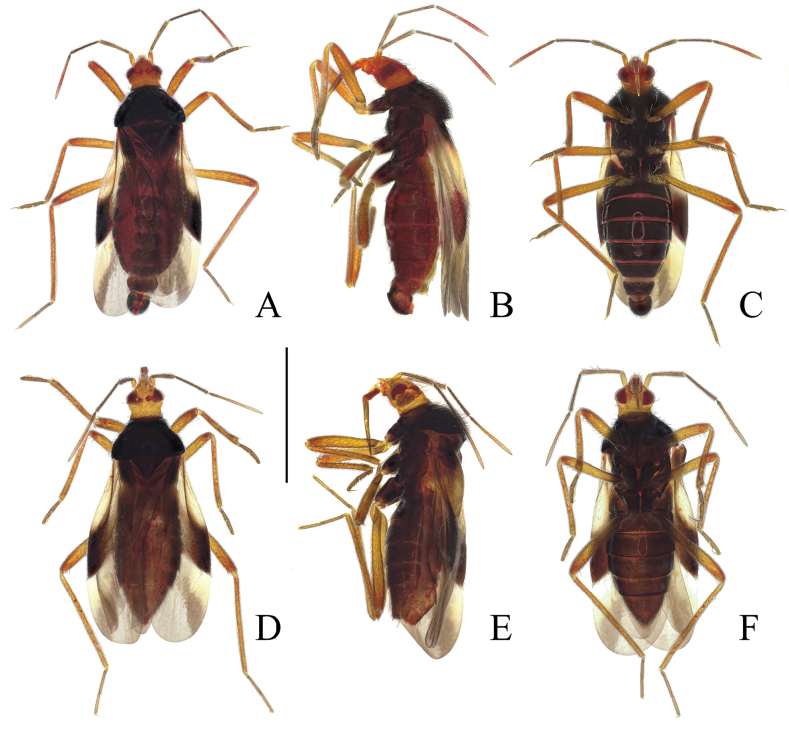
*Monteithophila hainanensis* sp. nov., habitus. **A**. Male, holotype, dorsal view; **B**. Same, lateral view; **C**. Same, ventral view; **D**. Female, paratype, dorsal view; **E**. Same, lateral view; **F**. Same, ventral view. Scale bar: 1 mm.

**Figure 2.**
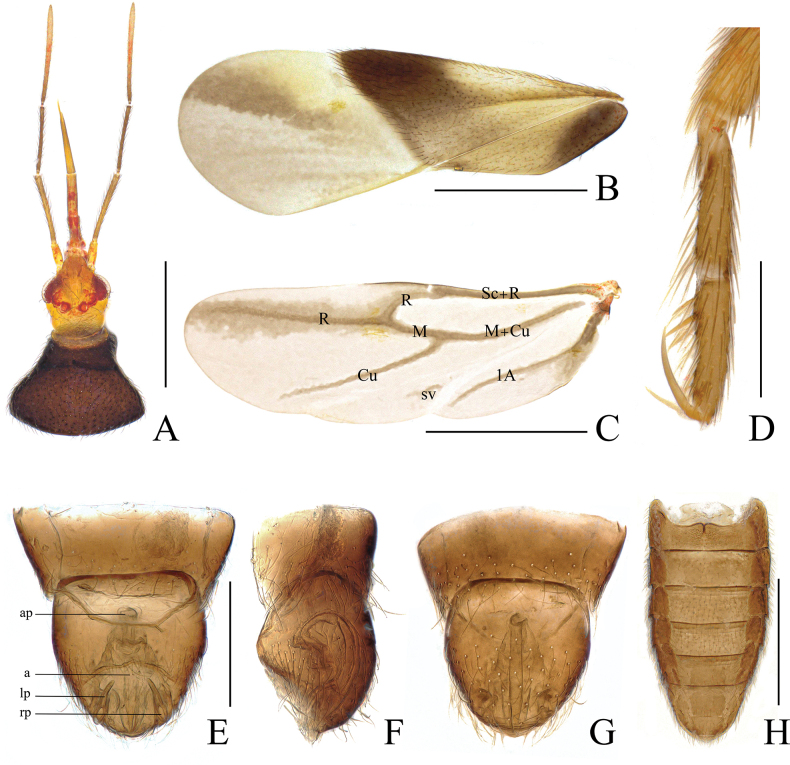
*Monteithophila hainanensis* sp. nov., diagnostic morphological characters. **A**. Head and pronotum, dorsal view; **B**. Hemelytron, dorsal view; **C**. Hind wing, dorsal view; **D**. Mid tarsi, lateral view, showing strong asymmetry of claws; **E**. Male genitalia, dorsal view; **F**. Same, lateral view; **G**. Same, ventral view; **H**. Female abdomen, dorsal view. Abbreviations: a = acus; ap = articulatory apparatus; lp = left paramere; rp = right paramere. Scale bars: 0.5 mm (**A, B, C, H**); 0.1 mm (**D–G**).

**Figure 3.**
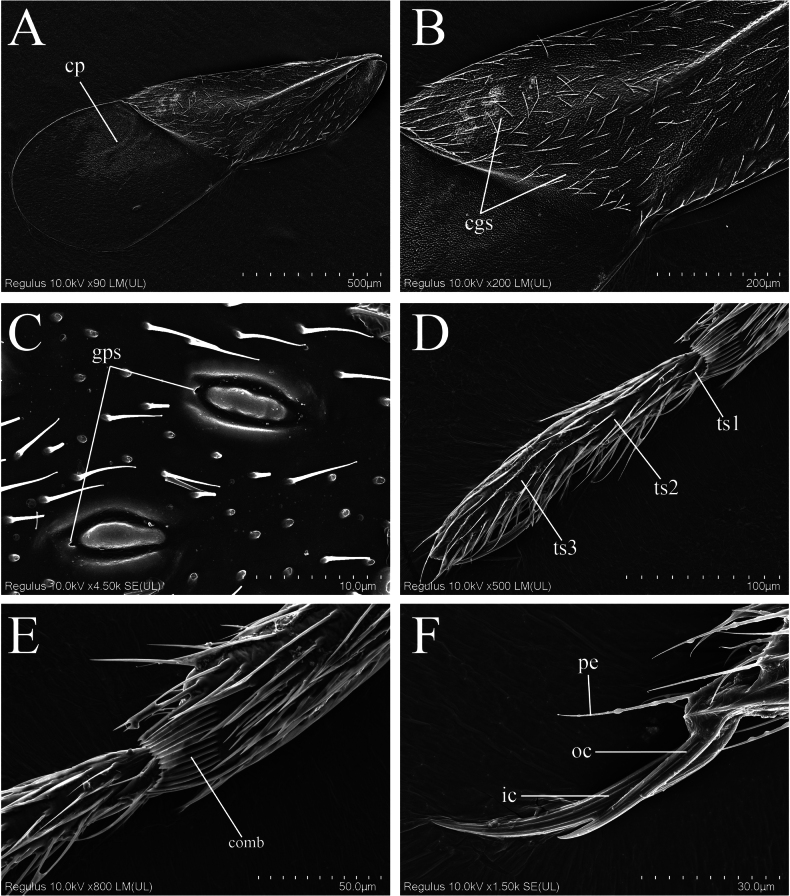
Scanning electron micrographs of *Monteithophila hainanensis* sp. nov. **A**. Hemelytron, dorsal view, showing corial process; **B**. Hemelytron, dorsal view, showing distribution area of corial glands; **C**. Corial gland, dorsal view, showing gland pore; **D**. Fore tarsus, lateral view; **E**. Apex of fore tibia, lateral view, showing comb; **F**. Claws of mid leg, showing strong asymmetry of claws, and parempodia. Abbreviations: cgs = corial glands; cp = corial process; gps = gland pores; ic = inner claw; oc = outer claw; pe = parempodia; ts1 = first segment of tarsus; ts2 = second segment of tarsus; ts3 = third segment of tarsus.

**Figure 4.**
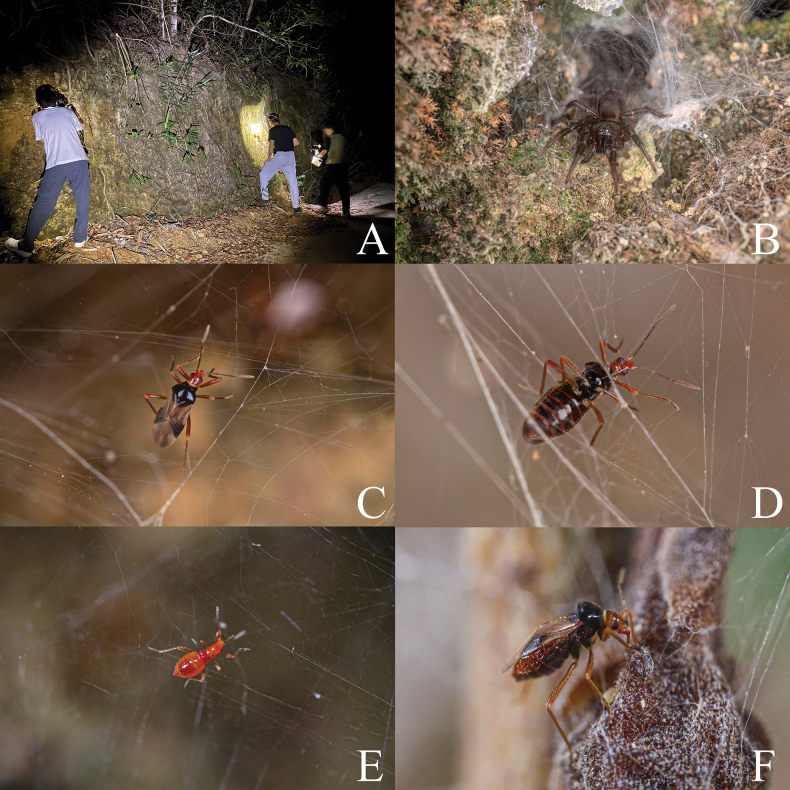
Habitats, spider host and living individuals of *Monteithophila hainanensis* sp. nov. **A**. Habitat in type locality; **B**. Spider host, *Spinathele* sp.; **C**. Adult female of *Monteithophila hainanensis* sp. nov., dorsal view; **D**. Same, ventral view; **E**. Nymph of *Monteithophila hainanensis* sp. nov.; **F**. Adult female of *Monteithophila hainanensis* sp. nov. feeding on mites on the spider web.

Figs 1, 2, 3, 4 Chinese vernacular name: 海南蒙丝蝽

#### Type material.

***Holotype***: • ♂, China, Hainan, Sanya Dist. [天涯区], Tianya, Liuluo Canyon [六罗峡谷], 18.4310°N, 109.5040°E, c. 478 m, 8.vi.2025, leg. Haoyang Xiong, Zhuo Chen & Yang Ge (CAU, accession number: CAUHN-PLO1). ***Paratypes***: • 6 ♂♂, 3 ♀♀ and 5 nymphs, same data as for holotype (CAU, accession number: CAUHN-PLO1).

#### Diagnosis.

This species is recognized within the genus by the following combination of character states: body length 1.8–2.0 mm; head yellowish brown to reddish brown; thorax black with a blue tinge; fore major claws slightly longer than mid ones with no significant difference; apex of corium and base of clavus dark brownish black; external component of corial gland elliptical, without constriction at middle.

#### Description.

Macropterous male and female (Fig. 1). ***Coloration***. Generally yellowish brown to reddish brown. Eyes dark brownish black. Ocelli red. Antennal segment I and apex of segment IV paler; remaining segments darker. Labium yellowish brown, apex of labial segment III and segment IV paler. Thorax black with a blue tinge, pronotal collar light brown. Legs yellowish brown; coxae dark brownish. Hemelytra light brown; apex of corium and base of clavus dark brownish black; membrane brownish black, outer half with dark spots, veins dark brown. Hind wings brownish black, slightly darkened near vein R; veins dark brown (Fig. 2C). Abdominal segments dark brownish black.

***Vestiture***. Body glossy, covered with short, semi-erect, simple setae. Head covered with sparse setae; antennae clothed with dark setae; first and second segments of labium covered with short, sparse setae. Pronotum densely covered with semi-erect, uniformly long setae; scutellum bearing sparse semi-erect setae. Legs covered with dense setae, apical part of femora relatively sparse; apex of fore tibia with a tuft of hairs representing an incipient grasping organ and with a comb for cleaning the antennae (Fig. 3E). Corium and clavus with subreclining setae (inclination angle of semi-erect setae on body surface ranges from 30° to 60°, while included angle between setae on wings and wing surface measures less than 30°), denser setae on anterior and outer margins of corium; corium densely covered with recumbent short setae (Fig. 3B). Ventral side of abdomen with sparse setae, lateral margins bearing dense setae. Apex of male genital segment with semi-erect setae (Fig. 2G).

***Structure***. Head (Fig. 2A) nearly cylindrical, distinctly narrowed towards apex in dorsal view, length slightly exceeding width; tylus broad, widening towards truncated apex. Eyes remote from collar. Ocelli small, with distance to eyes subequal to interocellar distance. Antennae slender; segment I thicker and shorter than others, slightly more than twice as long as tylus; ratio of lengths of antennal segments I:II:III:IV = 1:2.4:2.9:4.3 (male) or 1:2.6:2.7:4.1 (female) (see Table 1). Labium slender, apex extending beyond mid coxae; segment I short and broad; labial segment ratios I:II:III:IV = 1:1.8:2.5:3.8.

**Table 1.** Measurements (in mm) of *Monteithophila hainanensis* sp. nov.

Body part	Male (holotype)	Male (paratypes, *N* = 5)	Female (paratypes, *N* = 3)
Length of body	1.91	1.83–1.92	1.86–1.89
Length of head	0.32	0.30–0.33	0.31
Width across eyes	0.26	0.25–0.27	0.26–0.27
Interocular space	0.15	0.14–0.16	0.14–0.16
Length of antennal segment I	0.10	0.09–0.11	0.10–0.12
Length of antennal segment II	0.24	0.24–0.27	0.26–0.28
Length of antennal segment III	0.29	0.26–0.29	0.27
Length of antennal segment IV	0.43	0.43–0.46	0.38–0.41
Length of labial segment I	0.06	0.05–0.06	0.05–0.06
Length of labial segment II	0.11	0.11	0.12–0.13
Length of labial segment III	0.15	0.13–0.15	0.17
Length of labial segment IV	0.23	0.22–0.24	0.23–0.26
Maximum length of pronotum	0.35	0.31–0.36	0.38–0.41
Maximum width of pronotum	0.52	0.52–0.55	0.54
Length of fore femur	0.52	0.52–0.53	0.45–0.47
Length of fore tibia	0.46	0.44–0.46	0.44
Length of fore tarsus	0.24	0.22–0.24	0.23–0.24
Length of fore major claw	0.09	0.09–0.10	0.10
Length of mid femur	0.53	0.52–0.56	0.50–0.53
Length of mid tibia	0.50	0.48–0.50	0.52–0.55
Length of mid tarsus	0.21	0.19–0.21	0.22–0.24
Length of mid-major claw	0.08	0.08	0.07–0.08
Length of hind femur	0.62	0.62–0.62	0.67
Length of hind tibia	0.73	0.71–0.75	0.79–0.81
Length of hind tarsus	0.33	0.31–0.33	0.34–0.36
Length of hind major claw	0.07	0.07–0.08	0.07–0.08
Length of hemelytron	1.41	1.41–1.45	1.45–1.52
Length of abdomen	1.08	1.02–1.10	0.88–0.93
Maximum width of abdomen	0.52	0.48–0.53	0.45–0.51

Pronotum (Fig. 2A) trapezoidal, maximum width slightly less than twice the maximum length, strongly arched in lateral view, with distinct collar; lateral margins slightly curved inwardly at 1/3 from posterior margin; posterior margin slightly rounded. Scutellum with basal width slightly greater than its length, nearly flat.

Legs relatively slender. Femora slightly thickened at middle. Tibiae straight, subparallel; fore tibia with cleaning comb on medial surface at apex (Fig. 3E). Tarsi slender, 3-segmented, segment I extremely short, segments II and III subequal in length (Fig. 3D); fore and middle tarsi with three strong erect spines on segment III (Figs 2D, 3D); claws unequal in length (Figs 2D, 3F), weakly flattened, major (inner) claw very long, much longer than outer claw, ratio of outer to inner claws on all legs approx. 2:3, fore major claws slightly longer than mid ones with no significant difference, hind leg claws shorter; parempodia well-developed, setiform (Fig. 3F).

Hemelytra relatively broad, exceeding apex of abdomen, with anterior margin expanding. Costal margin of corium weakly sinuous, coriomembranal juncture nearly straight, well defined; corial process present sublaterally on membrane at juncture of corium and membrane (Fig. 3A). Corial glands numerous but small, mainly distributed on posterior half of corium (Fig. 3B); external component of corial gland elliptical, without constriction at middle, bearing elongate central mound, apex with gland pore (Fig. 3C). Membrane with three straight, longitudinal, free veins (Fig. 2B). Venation of hind wings as shown in Fig. 2C; Cu with distal free branch. Abdomen oval, length about twice as long as its maximum width. Mediotergites membranous. Sterna fully sclerotized; first sternite with notch at middle of upper margin, extending about 1/3 toward lower margin.

Male genitalia (Fig. 2E–G): pygophore short and broad, telescoped within abdominal segments VII and VIII, opening dorsad; parameres symmetrical, apex elongate, tubular, strongly curved near midpoint, apices exposed on dorsal surface, directed anteromedially; aedeagus chitinized, acus slender, apex acuminate.

Female genitalia (Fig. 2H): no ovipositor or copulatory tubes observed.

#### Etymology.

The specific epithet refers to Hainan, China, the province of the type locality of the new species.

#### Distribution.

**Figure 5.**
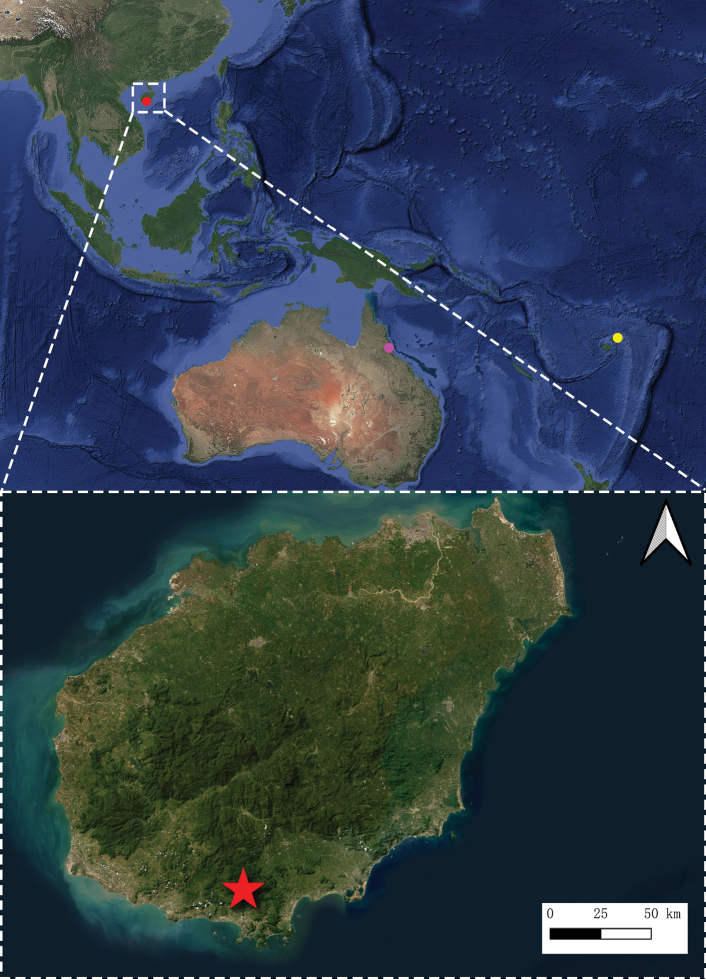
Known distribution of *Monteithophila*: purple dot = *M. queenslandana*; yellow dot = *M. fijiensis*; red dot and star = *M. hainanensis* sp. nov.

China (Hainan) (Fig. 5).

##### Key to species of *Monteithophila*

**Table:** 

1	Body length less than 2 mm; head uniformly colored, yellowish brown to reddish brown; fore major claws slightly longer than mid ones with no significant difference	***M. hainanensis* sp. nov**.
–	Body length more than 2.2 mm; head non-uniformly colored, mixed with castaneous and black; major claw of fore tarsus extremely long, significantly longer than that of mid tarsus	**2**
2	Body generally dark brown; length of head 0.9 times as long as interocular distance; eyes large	***M. queenslandana***
–	Body generally pale brown, with antennal segment I and legs much paler; length of head 1.45 times as long as interocular distance; eyes small	***M. fijiensis***

## Discussion

### Comparative notes

Compared with the two known species of *Monteithophila*, *M. hainanensis* sp. nov. has the following differences: it is smaller in size, with most individuals less than 2 mm, while the other two species are both over 2.2 mm in size; head uniformly colored, yellowish brown to reddish brown, without black or castaneous, distinctly different from the other two species; All three aforementioned species have tarsal claws of unequal length on all legs, but the new species has a relatively short major claw on the fore tarsus, with no obvious difference in length from the major claw on the mid tarsus; the external component of the corial gland is elliptical, without constriction in the middle. In addition, regarding the morphology of the genital segment, *M. hainanensis* sp. nov. also differs from the described characteristics of the known species. According to the description by Schuh et al. (2015), the male genital segment of the genus *Monteithophila* is telescoped within abdominal segments VII and VIII, with the endosoma baglike and inflatable. The *M. hainanensis* sp. nov. discovered in this study conforms to the characteristics of *Monteithophila* in terms of pygophore structure, pygophore opening direction, and paramere morphology (with the apex extending beyond the pygophore and directed anteromedially). However, anatomical observations under a light microscope revealed that the aedeagus of *M. hainanensis* sp. nov. is chitinized, and the acus is elongate and slender, which is similar to the aedeagus morphology of most species in Plokiophilidae (Fig. 2G). Therefore, we consider this species to be a distinctive member within *Monteithophila*. The specific taxonomic placement of this species and the genital characteristics of the genus require further investigation based on accumulated specimen materials in the future.

### Biological notes on *Monteithophila hainanensis* sp. nov.

Based on currently known distribution records, Plokiophilidae species are mostly distributed in low-latitude regions with warm temperatures and high humidity (Luo et al. 2021; Xiong et al. 2025). Prior to the implementation of field surveys, we hypothesized that plokiophilid bugs may be distributed on Hainan Island, southern China. Subsequently, we detected the presence of this family in Liuluo Canyon, approximately 30 km north of Sanya. On the soil slopes along the road without vegetation cover, many individuals of *Spinathele* sp. build webs and inhabit natural crevices, while *Monteithophila hainanensis* sp. nov. resides on these spider webs (Fig. 4A–E).

We examined dozens of webs, and nearly every web was inhabited simultaneously by adults and nymphs of various instars, with numbers ranging from 2 to 10 individuals. When observing the behavior of *M. hainanensis* sp. nov., it was found that they can feed on mites present on the spider webs (Fig. 4F). This is consistent with the feeding habits previously observed in *Embiophila sinica* Xiong, Chen, Li & Cai, 2025 and *E. africana* Carayon, 1974 (Carayon 1974; Xiong et al. 2025). During field surveys, we observed that the *M. hainanensis* sp. nov. aggregates around the spiders when their host is holding the prey; based on the results of this behavioral observation, we hypothesize that the species may also subsist on the prey items captured by the spiders. However, no observations have yet been made of them feeding on the host’s eggs, juveniles, or larvae.

Field observations of plokiophilid bugs showed that they prefer webs with well-developed vertical structures and are mostly active on the periphery of the webs. In the vicinity of *Spinathele* sp., the host of *M. hainanensis* sp. nov. (with the closest distance being no more than 10 cm), we also found two other ground-dwelling web-building spider species, *Cyriopagopus hainanus* Liang, Peng, Huang & Chen, 1999 and *Chilobrachys guangxiensis* Yin & Tan, 2000. However, after carefully inspecting multiple webs of these tarantulas, no plokiophilid bugs were found inhabiting with them. It is speculated that this is because the webs of the two aforementioned tarantula species are mat-shaped with a dense structure, which are not conducive to the movement of such bugs on them.

The known distribution of the three species of *Monteithophila* is shown in Fig. 5. *Monteithophila queenslandana* and *M. fijiensis* are both distributed in Oceania, while *M. hainanensis* sp. nov. represents the first record of this genus in the Oriental Region. The discovery of this species, following the records by Luo et al. (2021), Yin et al. (2023) and Xiong et al. (2025), constitutes the fourth record of Plokiophilidae in China. It is likely that southern China harbors more undiscovered plokiophilid species.

## Supplementary Material

XML Treatment for Monteithophila

XML Treatment for Monteithophila hainanensis

## Additional information

### Use of AI

No use of AI was reported.

### Author ORCIDs

Haoyang Xiong https://orcid.org/0000-0003-1086-5115

Zhuo Chen https://orcid.org/0000-0003-3393-0338

Hu Li https://orcid.org/0000-0001-8590-1753

Wanzhi Cai https://orcid.org/0000-0002-8620-0446

### Data availability

All of the data that support the findings of this study are available in the main text.
